# Regulation of adipogenesis by nucleotides

**DOI:** 10.1042/BST20253045

**Published:** 2025-06-30

**Authors:** Julia A. Pinette, Heather G. Bryant, Jacob W. Myers, Elma Zaganjor

**Affiliations:** 1 Department of Molecular Physiology and Biophysics, Vanderbilt University School of Medicine, Nashville, TN, U.S.A.; 2 Center for Stem Cell Biology, Vanderbilt University, Nashville, TN, U.S.A.; 3 Vanderbilt Digestive Disease Research Center, Vanderbilt University Medical Center, Nashville, TN, U.S.A.; 4 Vanderbilt Diabetes Research Center, Vanderbilt University Medical Center, Nashville, TN, U.S.A.; 5 Vanderbilt Center for Immunobiology, Vanderbilt University Medical Center, Nashville, TN, U.S.A.

**Keywords:** adipogenesis, lipid metabolism, metabolic regulation, mitochondria, purinergic signaling, white adipose tissue

## Abstract

The process by which multipotent cells commit to differentiate into distinct cell types, eventually forming functional tissues and organisms, has fascinated scientists for decades. Consequently, numerous studies have contributed to our understanding of how transcription factors and signaling molecules regulate differentiation. A growing area of interest in the field centers around the role of nutrients and metabolic pathways in cell fate determination. This review focuses on adipogenesis (also termed hyperplasia), the formation of adipocytes, which are key sensors of nutrient availability. We will examine recent findings that reshape our understanding of how nucleotide metabolism regulates adipogenesis.

## Introduction

The identification of fat tissue as a distinct anatomical and physiological entity dates back to ancient medical traditions, including the Ayurvedic concept of *meda dhatu* and Hippocratic discussions of corpulence as a disease state [1,2]. Scientific recognition of adipose tissue as a specific tissue type emerged during the nineteenth century, particularly with Arthur Hassall’s 1849 microscopic description of fat cells—then termed ‘fat vesicles’—marking the beginning of its formal anatomical classification [3,4]. Adipose tissue’s role was thought to be limited to energy storage and insulation until the mid-twentieth century, when the metabolic importance of adipose tissue began to emerge. In 1948, Shapiro and Wertheimer, in a review article, highlighted evidence that adipose tissue is innervated by sympathetic nerve fibers, possesses a dense network of capillaries, and that lipid storage and mobilization are highly regulated within the tissue [5]. This characterization introduced an important concept that adipose tissue is an active participant in organismal physiology.

Throughout the latter half of the twentieth century, the physiological relevance of adipose tissue was further revealed. A pivotal breakthrough came in 1994 when Friedman and colleagues identified leptin, the first known adipokine, while studying the *ob* (obese) gene, which encodes this hormone. Mutations in the leptin gene were found to cause severe obesity in mice [6]. This discovery redefined adipose tissue as a key endocrine organ, highlighting its role in regulating energy homeostasis, appetite, and metabolism.

Building on the importance of adipose tissue as an endocrine organ, another fundamental role is its ability to prevent hepatic and muscular lipotoxicity by safely storing excess nutrients. Excess energy intake, when not matched by energy expenditure, leads to the storage of surplus nutrients in adipose tissue, expanding fat depots. This expansion occurs through two mechanisms: hypertrophy, the enlargement of preexisting adipocytes through lipid accumulation, and adipogenesis, the formation of new adipocytes from the proliferation and differentiation of precursor cells, both of which are extensively characterized in the literature [7]. The balance between these mechanisms is governed by interactions between diet and genetics [8]. In obesity-mediated metabolic dysfunction, hypertrophy is linked to negative health effects, such as insulin resistance and fibrosis [9]. In contrast, increased adipogenesis is associated with improved insulin sensitivity and overall metabolic health [10]. Given the beneficial aspects of adipogenesis, many research studies have focused on identifying the regulatory mechanisms that govern this process, with the goal of mitigating the adverse effects of obesity.

### Transcriptional and signaling regulation of adipogenesis

**Figure 1:**
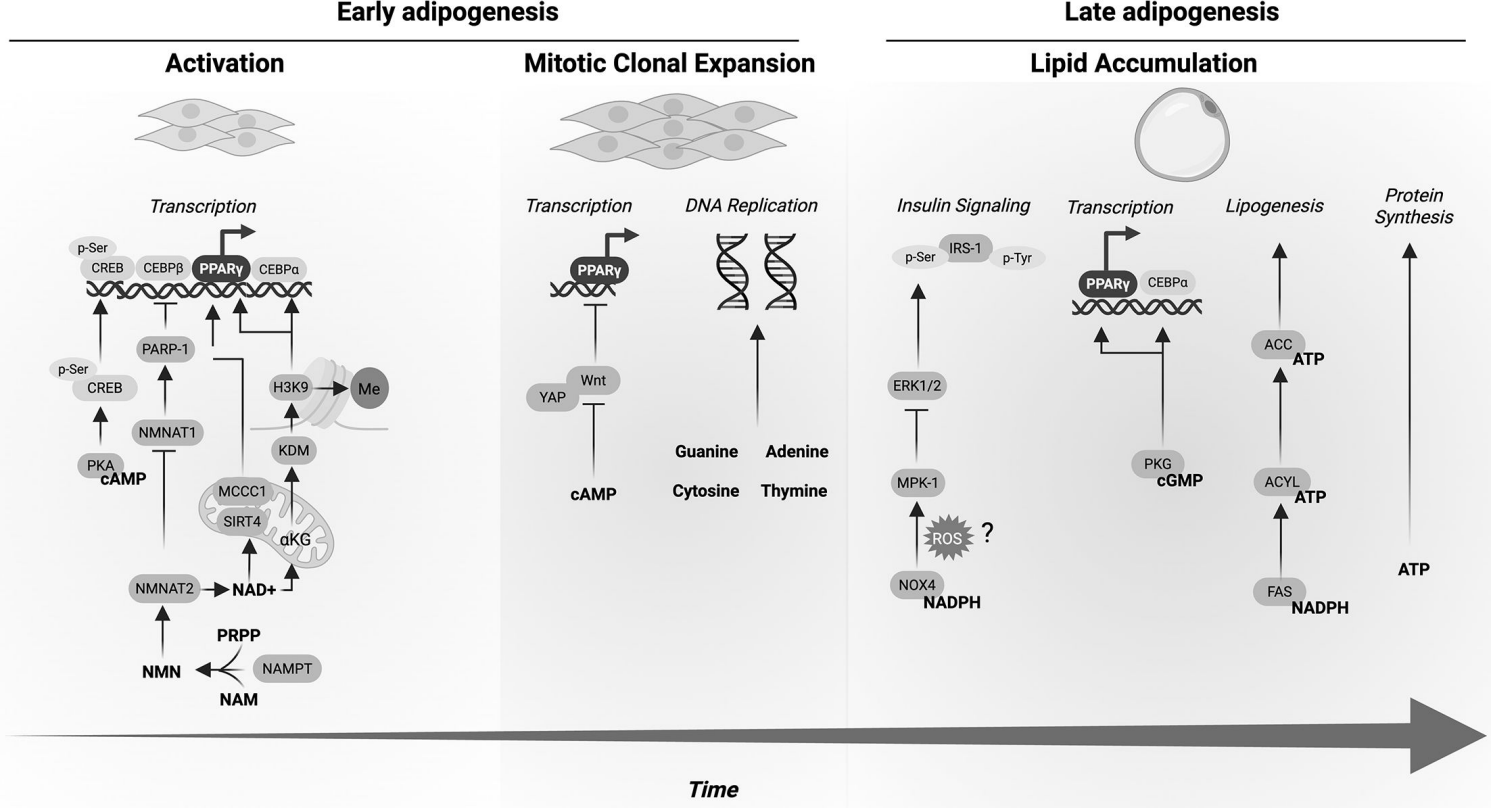
Regulation of adipogenesis by nucleotides. In early adipogenesis, cAMP regulates transcription through the activation of protein kinase A (PKA), leading to the phosphorylation of cAMP response element-binding protein (CREB). Nicotinamide mononucleotide (NMN) accumulation promotes the activity of nicotinamide mononucleotide adenylyl transferases (NMNAT2), resulting in the inhibition of anti-adipogenic enzyme polymerase 1 (PARP1) and the increased production of NAD^+^. NAD acts as a cofactor for sirtuin 4 (SIRT4), an enzyme that promotes mitochondrial fuel oxidation and promotes proliferator-activated receptor gamma (PPARγ). NAD accumulation results in increased α-ketoglutarate (αKG), leading to the demethylation of histone H3K9 and increased adipogenic transcription. During MCE, cyclic nucleotides and nucleobases inhibit Wnt and Yap signaling to increase adipogenic transcription and support DNA replication, respectively. In late differentiation, nucleotides act as cofactors for enzymes that promote insulin receptor signaling, enhance PPARγ and CEBPα, and support lipogenesis. ATP also provides energy to demanding processes like protein synthesis. Nicotinamide adenine dinucleotide phosphate (NADPH) may support adipogenesis by modulating the levels of reactive oxygen species (ROS). cGMP promotes adipogenic transcription via cGMP-dependent kinase (PKG) activation.

Adipogenesis involves two distinct phases that include mitotic clonal expansion (MCE) and lipid accumulation (Figure 1). These events are temporally co-ordinated by key transcriptional and signaling events. Preadipocytes, stimulated by insulin, glucocorticoids, or other proliferative agents [11,12], undergo multiple rounds of MCE [13,14] before terminal differentiation [15,16]. MCE is controlled at multiple levels, including regulation by transcription factors, DNA replication, cell cycle progression, and mitogenic signaling. The transcription factors c-Jun, c-Fos, and c-Myc play distinct, yet complementary, roles in promoting adipogenesis. c-Myc primarily facilitates DNA replication by up-regulating genes involved in DNA synthesis and chromatin remodeling. c-Jun and c-Fos, components of the AP-1 complex, drive cell cycle progression by enhancing the transcription of cyclins and other cell cycle regulators. Together, these factors contribute to mitogenic signaling, integrating extracellular growth cues to activate downstream pathways, such as mitogen-activated protein kinase (MAPK) and phosphoinositide 3-kinase (PI3K)/Akt, that support adipocyte proliferation and differentiation [17–19]. Cyclins and cyclin-dependent kinases regulate cell cycle progression, and their activity influences adipogenesis [20]. The expression of genes that regulate cell cycle progression is mediated by CCAAT/enhancer binding proteins (C/EBPs), C/EBPβ, and C/EBPδ, early transcriptional regulators.

C/EBPβ and C/EBPδ also support the lipid accumulation part of adipogenesis by activating peroxisome proliferator-activated receptor gamma (PPARγ). PPARγ exists in two isoforms, with PPARγ2 demonstrating increased fat specificity compared with PPARγ1. PPARγ induces C/EBPα expression, which then generates a positive feedback loop through a C/EBPα-mediated increase in PPARγ expression. This interaction between PPARγ and C/EBPα ensures the maintained expression of adipocyte-specific genes, for example, adiponectin [AdipoQ, a hormone that enhances insulin sensitivity and fatty acid oxidation (FAO)], fatty acid binding protein 4 (FABP4, facilitates intracellular lipid transport), and lipoprotein lipase (essential for hydrolyzing circulating triglycerides into free fatty acids for uptake and storage) [21].

Hormonal cues play a critical role in reinforcing this transcriptional network. In addition to these core factors, insulin—secreted by pancreatic β-cells in response to rising blood glucose levels—is a key inducer of adipogenesis, primarily acting through the insulin receptor and activating downstream signaling pathways involving IRS1/2, PI3K, and AKT. This cascade promotes glucose uptake and lipogenesis, supporting the growth and function of adipocytes. Insulin also stimulates the expression of key adipogenic transcription factors [22,23] and is important for maintaining adipocyte function and suppressing lipolysis [24]. Insulin induces sterol regulatory element-binding protein, promoting PPARγ expression and adipogenesis [21]. As insulin levels rise postprandially, they signal nutrient availability, triggering adipocyte differentiation to increase lipid storage capacity.

Glucocorticoids, including cortisol, signal to regulate adipogenesis through the glucocorticoid receptor (GR). Glucocorticoid-GR signaling induces CEBPβ, which subsequently activates PPARγ. However, the effects of glucocorticoids are complex. While insulin may enhance the pro-adipogenic effects of glucocorticoids, excessive glucocorticoid signaling can disrupt normal lipid metabolism and promote lipolysis in mature adipocytes [25,26].

Additional signaling pathways also influence adipogenesis. Bone morphogenetic proteins (BMPs) and Wingless-related integration site (WNT) signaling have opposing functions in adipogenesis. For example, BMP4 signaling activates Sma and Mad related (SMAD) proteins that increase the expression of PPARγ [27]. In contrast, Wnt10b stabilizes β-catenin, suppressing the activity of PPARγ [28,29].

Finally, cytoskeletal remodeling supports morphological maturation of adipocytes. As preadipocytes differentiate, changes in cytoskeletal structure, including the disassembly of actin filaments and microtubules, facilitate the morphological changes required for adipocyte formation. Ras homolog family member A (RhoA) signaling promotes cytoskeletal tension, and disruption of RhoA activity can enhance adipogenesis, suggesting that cytoskeletal reorganization is an important early event in the differentiation process [30,31].

### Metabolic regulation of adipogenesis

Metabolic regulators also contribute to adipogenesis [32], co-ordinating adipocyte differentiation with the organism’s energy status. Through the pentose phosphate pathway (PPP), glucose promotes adipocyte maturation by generating cofactors essential for lipogenesis [33]. Branched-chain amino acids (BCAAs)—leucine, isoleucine, and valine—serve as a source of lipogenic acetyl-CoA through mitochondrial transamination and oxidation [34,35]. BCAA oxidation is regulated by the mitochondrial sirtuin, SIRT4, which enhances adipocyte differentiation by increasing PPARγ activity [36]. Nuclear acetyl-CoA levels modulate histone acetylation, and its reduced availability inhibits adipocyte differentiation [37]. These studies highlight the mechanisms by which nutrient oxidation supports adipogenesis.

## Nucleotides in adipogenesis

Nucleotides take on many functional roles: precursors for DNA/RNA synthesis, cofactors for metabolic redox reactions, and signaling molecules. Nucleotides are important for the synthesis of DNA and RNA, and as signaling molecules that promote cellular proliferation required to support adipogenesis. DNA replication is required for proliferation [38–40], and impairing DNA synthesis or regulators of DNA replication impairs adipogenesis [41–43]. Across cell types, glutamine supports DNA replication in proliferation, and glutamine-depleted culture medium impairs proliferation [44]. The addition of adenine, adenosine, inosine, and hypoxanthine overcomes glutamine depletion, restoring DNA synthesis and proliferation in fibroblasts [38]. In adipogenesis, inhibition of *de novo* purine and pyrimidine biosynthesis reduces cell number, suppressing adipogenesis, consistent with impaired MCE [45,46]. These studies suggest that nucleotide availability regulates proliferation in adipogenesis. Extracellular purines can also bind to receptors on the plasma membrane and induce signaling cascades in an autocrine or paracrine manner [47]. Nucleotides regulate many adipogenic processes, such as the induction of adipogenesis, MCE, and lipogenesis (Figure 1).

**Figure 2:**
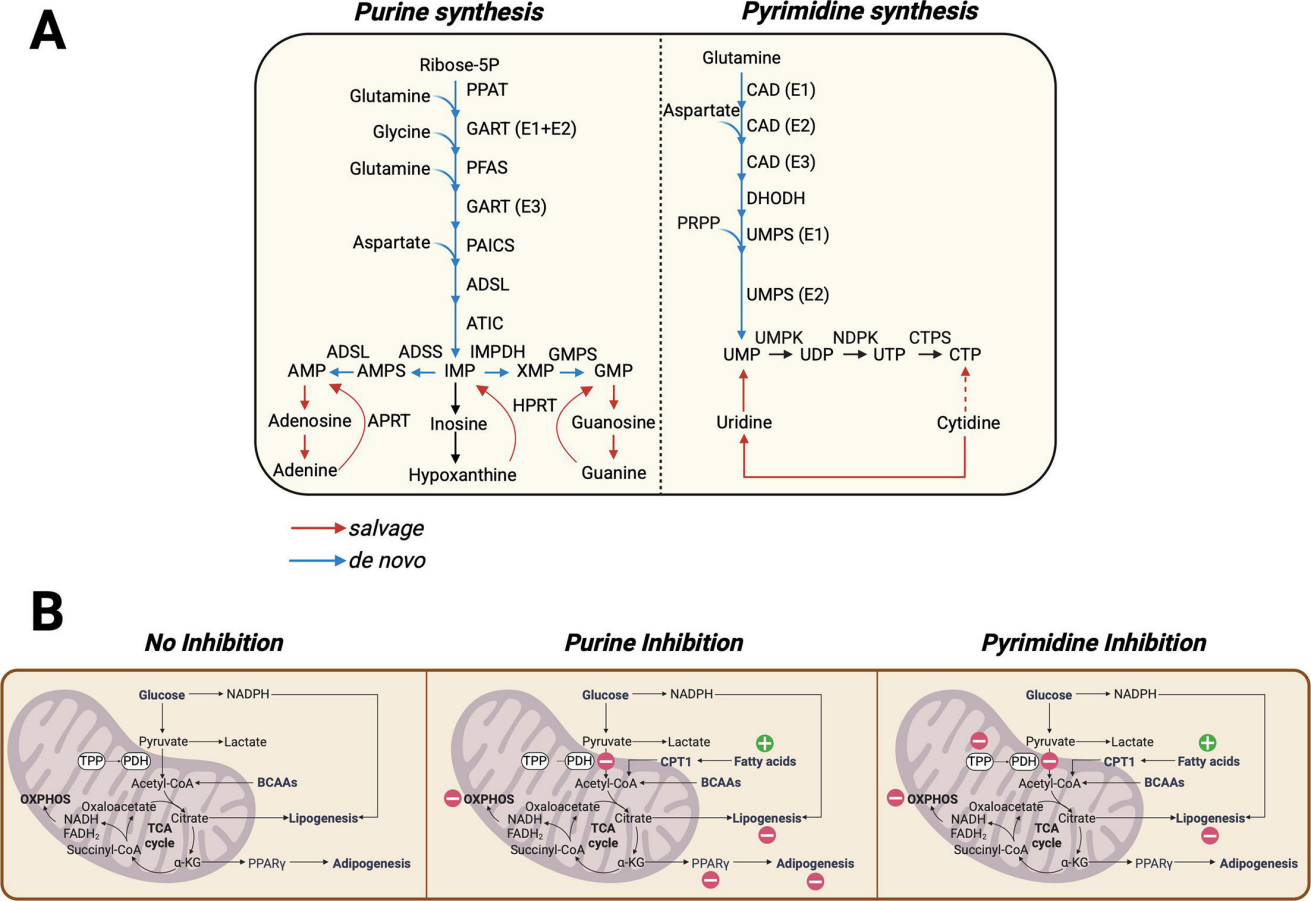
Nucleotide biosynthesis in adipogenesis. (**A**) Purine and pyrimidine biosynthetic pathways. Red arrows indicate the salvage pathway, and blue arrows indicate the *de novo* pathway. (**B**) Under conditions that stimulate adipogenesis, acetyl-CoA is derived from glucose and BCAAs, generating ATP via oxidative phosphorylation (OXPHOS), and performing lipogenesis using citrate from the TCA cycle. Under purine and pyrimidine inhibition, flux of pyruvate to acetyl-CoA is disrupted and FAO is elevated. Likewise, lipogenesis and OXPHOS are suppressed. Nucleotide inhibition results in arrested adipogenesis.

In quiescent or non-proliferating cells, the nucleotide salvage pathway is primarily responsible for maintaining intracellular nucleotide levels, whereas during times of growth, cells increase *de novo* nucleotide synthesis [48,49]. In *de novo* purine synthesis, phosphoribosyl pyrophosphate (PRPP) contributes a ribose sugar and a phosphate, while the addition of carbons and nitrogen from glycine, glutamine, aspartate, and N10-formyl-tetrahydrofolate ultimately leads to the generation of inosine monophosphate (IMP) (Figure 2A). IMP can then be converted to adenosine monophosphate (AMP) or to guanosine monophosphate (GMP) [50]. Through the salvage pathway, nucleosides and nucleobases can be recycled to produce AMP, GMP, or IMP. In the *de novo* synthesis of pyrimidines, the pyrimidine ring is first synthesized before the incorporation of PRPP to generate uridine monophosphate (UMP) [51,52]. UMP is then used to generate cytidine monophosphate (CMP) and thymidine monophosphate (TMP) [50,53]. Pyrimidines can also be generated via the salvage pathway, which involves the recycling of nucleosides. These nucleosides can be derived from the catabolism of nucleotides within the cell or obtained through extracellular uptake mediated by concentrative nucleoside transporters and equilibrative nucleoside transporters [54–57].

### cAMP

Cyclic adenosine monophosphate (cAMP) is synthesized from adenosine triphosphate (ATP) by the enzyme adenylate cyclase [58]. cAMP acts as a second messenger in signal transduction to mediate the effects of hormones, neurotransmitters, and inflammatory molecules to promote adipogenesis [59,60]. G-protein coupled receptors, activated during adipogenesis, enhance cAMP synthesis [61], promoting adipogenesis and altering adipocyte metabolism [62]. Elevated cAMP levels suppress the expression of anti-adipogenic factors like WNTs and Yes-associated protein (YAP), allowing the progression of adipogenesis after MCE [63–66]. cAMP also activates protein kinase A (PKA), promoting its nuclear translocation and allowing for the phosphorylation of cAMP response element-binding protein (CREB) [67,68]. Phosphorylated CREB then promotes the expression of the adipogenic transcriptional cascade [69,70].

### cGMP

cGMP, another cyclic nucleotide, mediates nitric oxide signaling [71], which regulates adipogenesis through distinct mechanisms from those of cAMP. Nitric oxide is synthesized from l-arginine by nitric oxide synthase enzymes [72]. NO diffuses freely across membranes and activates soluble guanylyl cyclase (sGC), leading to the production of cGMP [73]. Elevated cGMP levels activate PKG, up-regulating the expression of PPARγ, CEBPα, and genes in lipid metabolism, increasing triglyceride content in differentiating adipocytes [74].

### ATP

In adipogenesis, ATP is primarily generated through increased glycolysis and mitochondrial oxidation of BCAAs, which are up-regulated to meet the heightened energy demands. In addition, mitochondrial biogenesis and its enhanced activity also contribute to the elevated ATP supply [75]. ATP is not only an essential energy source for the biosynthetic processes involved in adipogenesis but also functions as a key cofactor for various enzymes and as a signaling molecule that modulates adipogenic processes [76]. Protein translation is one of the most energetically demanding processes [77], and adipocytes display a two-fold increase in protein levels during terminal differentiation, demonstrating a need for ATP [78]. ATP is required as a cofactor by several enzymes involved in *de novo* lipogenesis, including ATP citrate lyase (ACLY) and acetyl-CoA carboxylase, both of which are central to lipid biosynthesis and adipocyte maturation [79]. Finally, ATP can also regulate adipogenesis through purinergic signaling via the P2Y receptor, for example [76].

### NADPH

Nicotinamide adenine dinucleotide phosphate (NADPH) is generated through several pathways in adipogenesis, including the oxidative phase of the PPP [80], malic enzyme, which produces NADPH while converting malate to pyruvate, and isocitrate dehydrogenase, which generates NADPH during the conversion of isocitrate to α-ketoglutarate. A key function of NADPH is its role as an essential cofactor for lipogenesis [33]. Additionally, NADPH contributes to maintaining redox homeostasis by supporting glutathione and thioredoxin production, which are critical for mitigating ROS during adipogenesis [81].

### NAD

Nicotinamide adenine dinucleotide (NAD^+^) plays a key role in redox reactions, acting as a cofactor for various enzymes that regulate metabolism and transcription [82]. NAD^+^ is salvaged from nicotinamide (NAM), nicotinic acid, and nicotinamide ribose and is also generated *de novo* from tryptophan. In the NAD salvage pathway, NAM phosphoribosyltransferase (NAMPT) catalyzes the conversion of NAM into nicotinamide mononucleotide (NMN) using 5′-PRPP, which is then converted to NAD^+^ by nicotinamide mononucleotide adenylyl transferases (NMNATs) [83].

NAMPT not only aids in reprogramming metabolism in adipogenesis, promoting both glycolysis and mitochondrial respiration, but also regulates adipogenesis through epigenetic modifications. By increasing NAD^+^ levels and enhancing α-ketoglutarate availability, NAMPT promotes demethylation of histone H3K9 and increases the transcription of adipogenic transcription factors PPARγ and CEBPα [84].

NMNATs exhibit differential subcellular localizations: NMNAT-1 is nuclear, NMNAT-2 is cytoplasmic, and NMNAT-3 is mitochondrial. The synthesis of NAD^+^ in different cellular compartments has significant effects on adipogenesis. Knockdown of NMNAT1 promotes adipogenesis and lipid accumulation by decreasing the activity of poly(ADP-ribose) polymerase 1 (PARP1) [85], an inhibitor of CEBPβ [86]. NMNAT2 is up-regulated early in adipogenesis and supports differentiation by both limiting substrate availability for nuclear NMNAT1 and providing NAD^+^ to support increased glucose metabolism [85]. Additionally, increased mitochondrial NAD^+^ may increase substrate availability for SIRT4, a positive regulator of PPARγ in early adipogenesis [36]. In contrast, reduced nuclear NAD^+^ down-regulates SIRT1, a negative adipogenic regulator [87].

## Cross-talk between mitochondrial metabolism and nucleotides in the regulation of adipogenesis

Given the significance of nucleotides in adipogenesis and the therapeutic use of nucleotide biosynthesis inhibitors, several studies evaluated how blocking nucleotide biosynthesis altered cellular metabolism and the effect thereof on adipogenesis. First, Shinde and Nunn showed that pyrimidine and purine metabolites are elevated during adipogenesis. Blocking purine or pyrimidine biosynthesis blocked adipogenesis by suppressing the PPARγ−C/EBPα transcriptional program [88] (Figure 2B). While purine depletion has been shown to block the mechanistic target of rapamycin complex 1 (mTORC1) activation in proliferating cells, it had no effect on mTORC1 activity in adipogenic models [88–90]. Rather, inhibition of purine biosynthesis activated AMP-activated protein kinase (AMPK), although the phosphorylation of its substrates was not induced. Furthermore, the expression of lipogenesis and lipolysis genes was reduced, likely due to the suppression of PPARγ [88]. Importantly, interfering with purine availability through genetic deletion of inosine monophosphate dehydrogenase 2 disrupted adipose expansion *in vivo* [91].

Next, the Ben-Sahra group has highlighted that pyrimidines maintain pyruvate oxidation and the tricarboxylic citric acid (TCA) cycle in mitochondria through the regulation of pyruvate dehydrogenase (PDH) to support *de novo* lipogenesis and adipocyte differentiation [92]. Importantly, the enzyme thiamine pyrophosphate kinase 1 (TPK1), which is crucial for synthesizing thiamine pyrophosphate (TPP) from thiamine, was found to be regulated by pyrimidine concentrations [92] (Figure 2B). This contrasts with previous assumptions that TPK1 predominantly uses ATP for this reaction, revealing that the pyrimidine UTP—and not purines or other pyrimidines like CTP—is key in limiting cellular TPP synthesis [92–94]. Given that TPP is an essential cofactor for PDH, the enzyme that converts pyruvate into acetyl-CoA, UTP indirectly regulates pyruvate catabolism [92]. Through this mechanism, UTP significantly contributes to modulating metabolic processes vital for energy homeostasis and cell growth. When UTP is depleted (either by inhibiting pyrimidine synthesis with small molecules like brequinar or by knocking down genes in the pyrimidine pathway), PDH activity is impaired, and less acetyl-CoA is generated, thereby curtailing the substrate availability for fatty acid synthesis [92]. Furthermore, inhibition of pyrimidine biosynthesis increases FAO, suggesting compensation for the energy shortfall due to impaired pyruvate processing [92]. These findings are further supported by a study that showed that blocking purine or pyrimidine biosynthesis decreased glucose utilization for mitochondrial oxidative phosphorylation (OXPHOS) while increasing FAO [45] (Figure 2B). Inhibition of FAO in the presence of purine or pyrimidine biosynthesis inhibitors restored adipogenesis and mitochondrial activity [45].

Nucleotide-mediated activation of mitochondrial metabolism also drives citrate production. Citrate exported from the mitochondria into the cytosol is cleaved by ACLY to generate acetyl-CoA, a key substrate for *de novo* fatty acid synthesis, as well as histone acetylation [37]. The availability of acetyl-CoA directly influences lipid accumulation in differentiating adipocytes and modulates gene expression through epigenetic mechanisms that support the adipogenic transcriptional program. As such, ACLY-knockout results in repressed expression of PPARγ, adiponectin, and FABP4 [95]. Beyond histone acetylation, other epigenetic modifications, such as histone and DNA methylation, are also influenced by cellular metabolism and contribute significantly to adipogenesis. For instance, the methyl donor S-adenosylmethionine (SAM), derived from ATP and methionine, is required for both DNA and histone methylation and has been shown to regulate adipocyte gene expression. SAM enhances PPARγ expression and promotes lipid accumulation, partly through the suppression of the Wnt/β-catenin and Hedgehog signaling pathways [96]. Moreover, dynamic changes in histone methylation at key regulatory loci—such as demethylation of H3K27me3 and enrichment of H3K4me3—have been shown to facilitate transcriptional activation of adipogenic genes during differentiation [97,98]. DNA methylation also plays a repressive role early in adipogenesis, with demethylation at promoters of Cebpα and Pparγ correlating with their transcriptional activation [99]. Together, these studies reveal that nucleotide availability is not only essential for sustaining mitochondrial metabolism and biosynthetic processes but also critically governs the transcriptional and epigenetic networks that drive adipocyte differentiation and lipid accumulation.

## Conclusion

In summary, the process of adipogenesis is orchestrated through a complex interplay of transcriptional regulation, signaling pathways, and metabolism. Metabolic regulators, including the PPP and the metabolism of BCAAs, integrate the energy status of the cell with adipocyte differentiation. Likewise, nucleotides play significant signaling roles beyond their canonical functions in DNA and RNA synthesis, including regulating critical processes such as lipogenesis (Table 1). Recent findings have shown that pyrimidine and purine levels distinctly affect TCA cycle and glycolytic intermediates, with pyrimidine depletion impairing pyruvate processing and purine depletion altering mitochondrial function.

**Table 1:** Roles of nucleotide metabolism in adipogenesis: Summary of critical mechanisms by which nucleotides modulate adipogenesis.

Metabolite/metabolic pathway	Regulatory role	Effect on adipogenesis
*De novo* purine biosynthesis	Generates IMP used to produce AMP and GMP.	Inhibition reduces adipocyte proliferation and suppresses PPARγ, impairing adipogenesis [45,46,88].
*De novo* pyrimidine biosynthesis	Produces UMP for CMP and TMP synthesis; regulates TCA cycle via UTP and TPP.	Pyrimidine depletion reduces pyruvate oxidation, impairs lipogenesis, and shifts cells toward FAO [92].
Pentose phosphate pathway	Generates NADPH and ribose sugars for nucleotide synthesis and lipogenesis.	Enhances fatty acid synthesis during adipocyte maturation [33].
ATP	Provides energy and cofactors for biosynthetic processes and purinergic signaling.	Required for lipogenesis, protein translation, and adipocyte differentiation [76,78].
NADPH	Essential cofactor for lipogenesis; maintains redox homeostasis during differentiation.	Supports lipid biosynthesis and mitigates ROS in adipocytes [33,80].
NAD^+^	Acts as a cofactor for redox reactions and regulates epigenetic modifications.	Promotes metabolism through NAMPT and NMNAT activity; supports PPARγ and CEBPα expression via histone remodeling [84].
cAMP	Second messenger generated by adenylate cyclase; activates PKA and CREB.	Promotes adipogenic transcriptional cascades, suppresses anti-adipogenic factors like WNTs, and supports differentiation [60–70].
cGMP	Second messenger generated by NO-sGC signaling; activates PKG.	Up-regulates lipid metabolism genes and PPARγ/CEBPα expression, increasing triglyceride content in adipocytes [71–73].
Thiamine pyrophosphate (TPP)	Cofactor for PDH regulated by UTP concentration via TPK1 activity.	UTP controls acetyl-CoA production through PDH, modulating fatty acid synthesis [92]

CREB, cAMP response element-binding protein; FAO, fatty acid oxidation; NADPH, nicotinamide adenine dinucleotide phosphate; PDH, pyruvate dehydrogenase; PKA, protein kinase A; PPARγ, proliferator-activated receptor gamma; PKG, cGMP-dependent kinase; ROS, reactive oxygen species; TPP, thiamine pyrophosphate; TPK1, thiamine pyrophosphate kinase 1.

Disrupting nucleotide biosynthesis arrests adipogenesis, which may be detrimental to metabolic health given adipogenesis’ protective effects [10]. However, recent work has shown that *in vivo* pharmacological inhibition of purine biosynthesis blocks fat tissue expansion by decreasing caloric intake and increasing energy expenditure through skeletal muscle thermogenesis [100]. This may confer protection against the adverse effects of chronic caloric surplus, such as insulin resistance and ectopic lipid accumulation. Several small-molecule inhibitors of purine and pyrimidine metabolism are currently in clinical use for treating rheumatoid arthritis, Crohn’s disease, ulcerative colitis, and numerous cancers [101–108]. The effects of these drugs on body weight extend beyond their intended therapeutic use as immunosuppressants and anticancer agents, and therefore, further research is needed to understand the risks and benefits associated with their use. Additional studies are needed to examine the specific cell type-dependent effects of purine and pyrimidine depletion on cellular physiology.

**Perspectives**

- Adipogenesis is an important protective mechanism that allows for the safe storage of excess nutrients. Given the alarming rise in obesity, there is much interest in understanding the regulation of this process. Here, we summarize the roles of nucleotides in adipogenesis.
- Beyond supporting DNA replication and cellular proliferation, studies have also shown the role of nucleotides in the regulation of signaling and transcription mechanisms required for adipogenesis. More recently, purines and pyrimidines were found to be essential for metabolic reprogramming that stimulates adipogenesis.
- Inhibitors of nucleotide biosynthesis are used to treat various disease states. Future studies should investigate how the use of these inhibitors affects metabolic health, particularly whether there are risks or benefits associated with their use in obese or cachectic individuals.

## Abbreviations

ACLY: ATP citrate lyase
BCAAs: branched-chain amino acids
BMPs: bone morphogenetic proteins
C/EBPs: CCAAT/enhancer binding proteins
CREB: cAMP response element-binding protein
FABP4: fatty acid binding protein 4
FAO: fatty acid oxidation
GR: glucocorticoid receptor
MCE: mitotic clonal expansion
NADPH: nicotinamide adenine dinucleotide phosphate
NAM: nicotinamide
NAMPT: nicotinamide phosphoribosyltransferase
NMATs: nicotinamide mononucleotide adenylyl transferases
NMN: nicotinamide mononucleotide
PARP1: polymerase 1
PDH: pyruvate dehydrogenase
PKA: protein kinase A
PKG: cGMP-dependent kinase
PPARγ: proliferator-activated receptor gamma
PPP: pentose phosphate pathway
PRPP: phosphoribosyl pyrophosphate
ROS: reactive oxygen species
RhoA: Ras homolog family member A
SIRT4: sirtuin 4
SMAD: Sma and Mad related proteins
TPK1: thiamine pyrophosphate kinase 1
TPP: thiamine pyrophosphate
WNT: wingless-related integration site
YAP: yes-associated protein
mTORC1: mechanistic target of rapamycin complex 1
sGC: soluble guanylyl cyclase
